# On Some Points Connected with Cholera

**Published:** 1889-08-17

**Authors:** 


### On Some Points Connected with Cholera.

### I.—ITS DEFINITION AND SYMPTOMS.

Cholera has been shortly defined by various authors in different terms. The following definition differs somewhat from those found in textbooks, but it is nevertheless in accordance with the characteristics of the disease:

"A disease always present in some tropical countries, and frequently becoming epidemic; generally characterised by purging and vomiting, usually of a material resembling water in which rice has been boiled; commonly accompanied by cramps; and frequently resulting in suppression of urine, the non-secretion of bile, and subsequent collapse."

Now, it was stated above that the definition differs from that of other authors. It will be observed that the ordinary symptoms are qualified by the observation that the disease is generally characterised by purging and vomiting, usually of a material resembling rice water, commonly accompanied by cramps, and frequently resulting in suppression of urine. As the paper progresses, it will be shown that such qualifications are right and necessary if we are to arrive at a knowledge of the real characteristics of the disease. It will be first necessary to detail the symptoms of a typical case of cholera.

Cholera is usually preceded by *malaise* and painless diarrhœa, which may extend from a few hours to ten days, or even longer. During the initial period of the malady, when remedies would probably be efficacious, sufferers often neglect to apply. The seizure very frequently occurs during the early morning hours, when the atmosphere is most chilly and the vital powers least resistant; an observation as regards other diseases at least as old as Shakespeare. It has also been observed that natives of India often suffer soon after a full meal, their custom being to distend their stomach to the utmost, and so avoid the trouble of cooking more than once or twice daily. The first symptom is spasmodic griping, followed by vomiting and purging, first of the contents of the stomach and intestines, and then of large quantities of a turbid whitish, almost odourless, fluid, with flocculent atoms floating on it, found under the microscope to be epithelum casts from the mucous membrane of the bowels. These rice-water stools may amount to fifteen or twenty in the course of a few hours, and at first they are discharged with great force, and are followed by a sense of relief, although by a feeling of exhaustion at the pit of the stomach. Vomiting may be equally frequent, and the ease with which the cholera-stricken vomit is often remarkable; the material sometimes passing up as if by simple regurgitation. Simultaneously with the vomiting and purging, or very soon after, severe cramps come on, sometimes commencing on the fingers and toes, sometimes alternating with tingling and rapidly extending to the calves, thighs, and abdominal muscles. With all this there is at first scanty high-coloured, often albuminous urine deficient in urea, and then total suppression of urine; a burning sensation and feeling of constriction at the epigastrum, which is sensitive to pressure; a white tremulous tongue, and a bitter taste in the mouth; great thirst, little or no saliva being secreted, and an urgent desire for cold drinks; a feeble pulse, but more frequent than normal, rising probably to 96, a cool skin, and no fever, although the patient complains of heat and oppression, and prefers to be uncovered. Noises in the ears may also be complained of; there is much restlessness, the patient often tossing about the bed. Lastly, a rapidly progressing exhaustion is evident. The case now stands on the verge of collapse. Should this succeed, the pulse becomes quicker, but hardly perceptible, the discharges cease, and so often do the cramps. The skin is covered with cold perspiration, has a sickly smell, and a bluish tinge. The nails and lips especially assume this appearance. The whole body shrinks and seems to wither, the genitals are retracted, and the skin of the fingers corrugated. The voice is faint and husky, and the tongue and expired air cold. The intelligence is ordinarily clear, but there is complete apathy as to the result. "Death," says Chevers, "is marked by a peculiar physiognomy, the body is of a bluish-grey, with collapsed face, hollow features, pointed tongue, deeply-sunken and closed eyes, although the patient still possess consciousness, and may be roused." Often two or three hours before death some return of heat in the scalp, forehead, or even over the chest may present. This is due to relaxation of minute arterial branches and onward flow of blood, and is usually a fatal sign.

In those cases which recover, the vomiting and purging gradually subside, the skin becomes warmer, the pulse fuller, the voice regains power, urine is again voided, colour appears in the stools, and the patient falls into a refreshing sleep. Even in apparently hopeless cases recovery may take place. So long as the patient has strength to vomit the case is not hopeless. But we can never be satisfied that the immediate danger is over till natural urine is secreted, and the average time of passing urine is 72 hours after seizure. There are, however, secondary results of cholera which are apt to prove dangerous. Ordinary fever may occur, attributable to simple reaction, and ordinary fever may be accompanied by cerebral symptoms. This is more likely to occur with Europeans than natives of India, and especially when much stimulants have been given. This fever may be mild, terminating in a few hours with an eruption of *roseola*, or nettle-rash. But the reactionary fever may be more severe, the urine again becomes scanty, deficient in urea, containing albumen, and uræmic poisoning occurs, with all the symptoms of the typhoid state. The elements of bile being also in the blood further complicate the case, and tend to the induction of delirium: sometimes a condition resembling apoplexy occurs suddenly; or occasionally convulsions. Lastly, persons who have previously suffered much from dyspepsia are liable to inveterate hiccup after cholera, rendering them unable to take any nourishment, depriving them of rest, and inducing a very exhausted condition.

Although the above may be regarded as a fair description of a typical case of cholera, there are, in fact, few diseases in which the symptoms, whilst retaining certain common characteristics, vary more. This variability of the disease was noticed on the first reputed invasion of cholera in 1832, when a Dr. Pennick pointed out how the epidemic in England differed from the disease in Russia. About the same time, Allan Webb asked in India, "Who can tell where cholera ends, and plague begins, or distinguish between certain forms of cholera and fever?" But long before this, Clarke had recorded an epidemic on the Coromandel Coast presenting all the symptoms of cholera except purging. Macpherson states that the disease is essentially the same, but the cholera of one season varies from that of another. Ziemssen remarks "almost every epidemic has its physiognomy." The late lamented Chevers stated, "Familiar as I was with the cholera of Calcutta, the disease which I treated during the greater part of 1874 was of a type altogether new to me—no two cases were precisely the same"; and again, "As the malarious poison produces all variations from a fatal remittent to the mildest ague, so with the cholera poison." Sir George Hunter also wrote, "As there are varying degrees of fever, so there are varying degrees of cholera. It may manifest itself by a slight looseness of the bowels, or as a sudden paralysis of the vaso-motor system."

Amongst other peculiar symptoms which have been noted in cholera, the following may be mentioned: A very slow pulse for some days or weeks antecedent to the attack. Sometimes the premonitory symptoms of *malaise* or diarrhœa do not occur. So often is this the case, that Surgeon-General Pringle considers there is usually no such stage. In most great outbreaks, at first, persons die suddenly from collapse without distinctive symptoms, often without vomiting or purging, or after one or two loose motions. This was particularly noticed in Bombay in 1883, while at Poonah cases assumed the form of the sweating sickness of olden days. In instances when death occurs from sudden collapse, without vomiting or purging, there may be violent cramps of the lower extremities. Chevers mentions two epidemics in Bengal having all the symptoms of cholera, excepting the stools being tinged with blood. C. A. Gordon mentions cases in which black vomit occurred, resembling that of yellow fever. A similar outbreak has been described as hæmorrhagic cholera, when the stools consisted of mucus or gelatinous fluid mixed with blood. Rice-water evacuations may be absent in the old and feeble, or rice water may be found in the intestines after death, none having been passed during life. Sometimes the stools are not turbid like rice water, but colourless like blood serum. Nitric acid sometimes gives a ruby-coloured reaction with rice-water stools, sometimes not, while in some cases cramps are absent; cramps and twitchings have been noticed without other symptoms. Also sensations of pins and needles, anæsthesia, or paralysis are occasionally present. Vomiting of worms has often been noticed during an attack. Dickson, speaking from his experience in the East, states : "An increased flow of urine is sometimes a symptom of epidemic cholera. Sometimes the urine, if passed, instead of albumen, contains sugar. Occasionally from cholera patients there is a peculiar sickening smell, likened by Murray to *arum maculatum*, or tainted fish. Cholera in its commencement may simulate an attack of ague, or it may terminate in fever, or there may be no fever. In females there is sometimes a sanguineous discharge when the person is not menstruating. The period and profundity of cholera is very variable, and the greater or less lividity of the countenance has given rise to such appellations as "blue" and "black" cholera. After death from cholera a remarkable contraction of voluntary muscles sometimes occurs, which has led to stories of persons being removed to the dead-house while yet alive. A tale is told of an European soldier being removed to the dead-house, where he recovered. A native sepoy sentry had been placed over the dead-house, who replied to the excited European's demands to be let out, "Na, na, sahib, tumara bockus na aya," which, being interpreted, is, "No, no, sahib, your box or coffin has not come." The spasmodic contortions seen after cholera death are due, it is believed, to *post-mortem* relaxation of arteries and onward flow of blood. Occasionally for the same reason heat of body obtains for a longer time than usual after death. Among the peculiar sequelæ of cholera are to be noted convulsions, persistent hiccup, nettle-rash, retention of urine, hæmorrhage, abscesses, and abortion in pregnant females. It is, therefore, scarcely matter of surprise that a disease presenting such diverse features should have been described under many names. Thus we have in the writings of European authors the terms cholera asiatica, asphyxia, algide, black, blue, dry, epidemic, endemic, febris remittens choleroidea, malarious, malignant, nervous, tetanic, nostras, pestiferous, hæmorrhagic, serous, simplex, spasmodic, sporadic, syncopal. There are also the terms cholerine, choleriform, choleroid disease, choleroid fever or sweating sickness, choleroid cholic, and white dysentery. All these are comparatively recent terms, called into use by the varying nature of the malady. The disease appeared under different phases, and both in India and Europe was described as it appeared; taking its second appellation from the most prominent characteristics.

Now the very name of cholera terrifies some people, and next to fever it is the most destructive of tropical maladies. There are no recent statistics to refer to, but in round numbers the mortality of a "cholera year" in India may be estimated at 100,000. Therefore it is not matter of surprise that when an epidemic "rushes as a storm o'er the astonished land, and strews with sudden carcases the earth," the greatest alarm should be experienced. Still, it is well to remember that even in the worst epidemics, attack is the exception and immunity the rule; for, as afterwards mentioned, fear is a powerful predisposing agent.

But for a disease so sudden, so diverse in character, and so destructive, there must be some other cause than fear, and the theories which have been suggested are numerous. The older writers attributed the malady to fatigue, want, unwholesome food, chills, etc., and did not look for any specific poison. Some half century back Dr. Tytler attempted to show that eating new or diseased rice was the cause. Very recently the assertion was revived, that there is a connection between eating fish with worms in them and cholera. Several kinds of fish consumed in India are known at certain seasons to be infested with worms and organisms similar to those reported by Koch in cholera excreta. The question was, however, investigated by the late Deputy - Surgeon - General Furnel, of Madras, who saw no reason to connect cholera with fish. It should, however, be recollected that a fish which is quite wholesome when absolutely fresh, may become otherwise if kept only a few hours in a hot, moist atmosphere from the formation of a *post-mortem* poison. Oysters have a particularly bad reputation in this respect. In Bombay, choleraic attacks after eating oysters are often heard of. But during cholera seasons any imprudence in diet is likely to excite an attack. That bad fish cannot be the cause is sufficiently evident, as the disease often occurs where the people rarely, if ever, see fish.

(*To be continued.*)

